# Chairside learning on undergraduate clinics: general dental and human disease themes

**DOI:** 10.1038/s41415-024-7676-1

**Published:** 2024-08-09

**Authors:** Philip A. Atkin, Anum Khan, Melanie L. Simms

**Affiliations:** Cardiff Dental Hospital and School, University Hospital of Wales, Heath Park, Cardiff, CF14 4XY, UK

## Abstract

**Introduction** Chairside teaching is an important part of dental undergraduate clinical education. Bedside teaching is well-reported in relation to undergraduate medicine but few publications relate to undergraduate dentistry.

**Aims and objectives** To investigate the experience and benefits from chairside teaching/learning in the clinical years of a five-year dental programme in a UK university. We asked about general dental topic learning as well as human disease (HD) learning.

**Materials and methods** An online survey gathered dental students' opinions on their recognition, knowledge and experience of chairside teaching/learning. We asked about clinics and clinicians and where they gained most from chairside teaching/learning. We encouraged free-text responses.

**Results** Altogether, 127 students took part (62% response). Response rates approximated 60% for all three years. In total, 93% felt that chairside teaching/learning helped to a moderate/great extent for general dental topics and 73% felt that chairside teaching/learning helped to a moderate/great extent for HD topics. Free-text comments revealed students valued chairside teaching/learning and from which grade/level of staff they learned most.

**Discussion and conclusion** Chairside teaching/learning is largely unreported and unrecognised in undergraduate education. Although not formally timetabled, enthusiastic staff with enough time to engage in chairside teaching can have a positive impact on student learning.

## Key points

- Chairside (bedside) teaching occurs in medical and dental undergraduate teaching and is under-recognised and under-reported but a valuable mode of teaching and learning.
- Chairside teaching will also occur in dental foundation training and is similarly valuable.
- Publication of this research will hopefully help teachers and learners recognise this valuable addition to undergraduate and postgraduate education.

## Introduction

Bedside teaching and learning have a long history in medicine. Over 2,000 years ago, in ancient Greece, Hippocrates was said to be the father of bedside teaching in medicine,^1^ and in seventeenth-century Europe, aspiring doctors travelled from across the globe to witness the physician Herman Boehave give clinical demonstrations from the patient's bedside.^2^ Historically in dental education, prior to universities taking over the formal delivery of dental education, most dentists learned their craft working as apprentices to established dentists, literally standing with them at the chairside.^3^^,^^4^

Sir William Osler (1849-1919) was one of the greatest promoters of bedside learning in medicine, famously declaring ‘medicine is learned by the bedside and not in the classroom'.^5^ It could be argued that the same is true of dentistry. Unlike in medical education, which is largely observational until the point of qualification, dental students gradually gain and develop their clinical skills until the point of graduation, where they are deemed competent to provide dental services to their patients without supervision.

Alcolado (2018) said ‘bedside teaching has the potential to be one of the most effective modalities in medical education…it can provide all the key elements known to be associated with effectual deep learning. It can be interactive, relevant, targeted, timely and encourage critical thinking skills'.^6^ Chairside learning for dental students with clinical supervisors is at least as important as bedside teaching for medical students from physicians and surgeons.

In modern dental education, much teaching and learning takes place in lecture theatres, seminar rooms and phantom head suites, as well as supervision and teaching on restorative dental education clinics (DEC) for treatment of patients. In most dental hospitals and schools, dental students are also attending consultant-led dental service clinics where patients present for diagnosis and treatment, having been referred by medical or dental practitioners (eg oral medicine and oral surgery/maxillofacial surgery clinics), as well as dental emergency clinics, where patients attend for examination, investigation and treatment of their acute dental problem. In both DEC and service clinics, there may be short opportunities for impromptu student teaching and learning, such as while patients are away from the dental chair having radiographs taken, and on their return during explanation of diagnosis and treatment planning.

## Aims and objectives

Earlier studies have identified opportunities for dental students to reinforce their learning in human disease (HD) because of the relative medical complexity of patients attending for dental care,^7^^,^^8^ and we wished to explore these themes further by surveying undergraduate dental students in Years 3-5 on their experiences of chairside teaching and learning in dental clinics, both in relation to general dental topics and also HD topics.

## Materials and methods

Following approval by the university dental school research ethics committee (DSREC 2209a), an online survey was used to anonymously collect students' responses to questions or statements (via tick-box or Likert-scale questions) and students' views (via free-text boxes) about their experience of chairside teaching and learning, both in relation to general dental topics, as well as those related to their knowledge and understanding of HD topics.^9^^,^^10^

In the introduction to the online survey, we explained what we meant by chairside teaching and learning:

- ‘Chairside teaching' can be defined as taking the opportunity for unplanned impromptu teaching and learning in topics raised as a result of the patient attending for examination and treatment.

Invitations with a URL link to the survey were emailed to each year group and posters were also displayed on student dental clinics, explaining the research and giving a QR code which linked to the survey. The survey was open for four weeks, with email reminders being sent after two and three weeks. The survey was entirely anonymous, other than asking students which year they were currently in, to allow analysis of the different groups separately. There were free-text boxes in each section of the questionnaire so that students could expand or clarify their responses. The survey was carried out during the second (spring) term, when third-year dental students have had reasonable experience of treating patients on clinics, and all students are well-established in their dental undergraduate programme.

The survey asked students about their experience of ad hoc chairside teaching episodes to see where they felt they were most beneficial, and where there may be missed opportunities that could be promoted in the future to enhance student learning.

Participation in the study was entirely voluntary and participants were advised they were under no obligation to participate. If they chose to participate by completing the questionnaire, then they also consented to enter the study. Participants remained anonymous and could choose to discontinue completion of the survey at any time.

## Results

The response rates each clinical year were: Bachelor of Dental Surgery (BDS) 3 = 64% (n = 42), BDS 4 = 64% (n = 44) and BDS 5 = 58% (n = 42). The overall response rate was 62% (n = 127).

The questionnaire had four main sections. The initial questions in the survey asked students if they recognised that there were opportunities for chairside teaching/learning in the clinical dental programme and if they had experienced chairside teaching/learning during their course. Overall, 98% of respondents stated they recognised the opportunity and had experienced chairside teaching/learning.

### Section 1: In which clinics was there most experience of chairside teaching/learning?

**Fig. 1:**
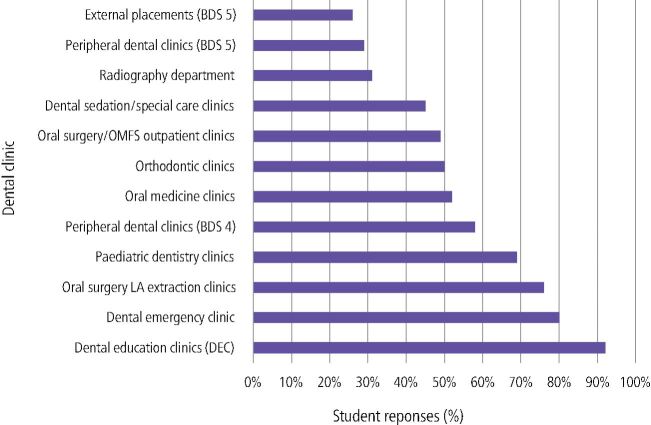
Which clinic has provided you with most experience of chairside learning in any dental topic?

In relation to any dental topic, students experienced most chairside learning in the restorative DEC clinic (92%). This was followed by the dental emergency clinic (80%), oral surgery local anaesthetic (LA) extraction clinic (76%) and paediatric dentistry clinic (69%) (Fig. 1). Free-text comments included:

- ‘All clinics. You learn a lot through experience and lots of differing clinicians'.

**Fig. 2:**
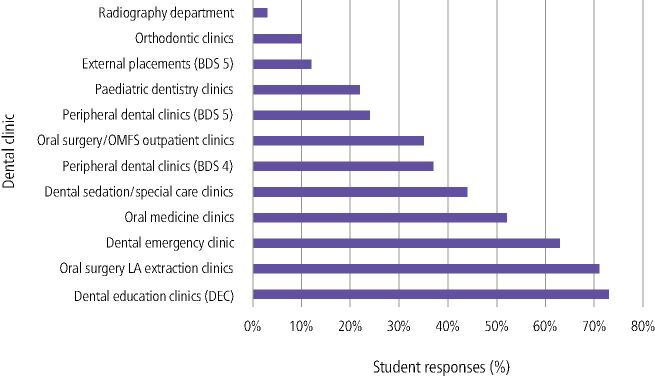
Which clinic has provided you with most experience of chairside learning related to human disease?

In relation to HD teaching, the students most commonly reported the restorative DEC clinic (73%) followed by oral surgery LA extraction clinic (71%), dental emergency clinic (63%) and oral medicine clinic (52%) in providing chairside learning (Fig. 2). Free-text comments included:

- ‘[Oral surgery] especially questioning on medications and what they might be being used for pre-op and the effects that the use of these meds will have on the XLA [extraction under local anaesthetic] post-op management'.

### Section 2: Which level of staff engaged most in chairside learning?

**Fig. 3:**
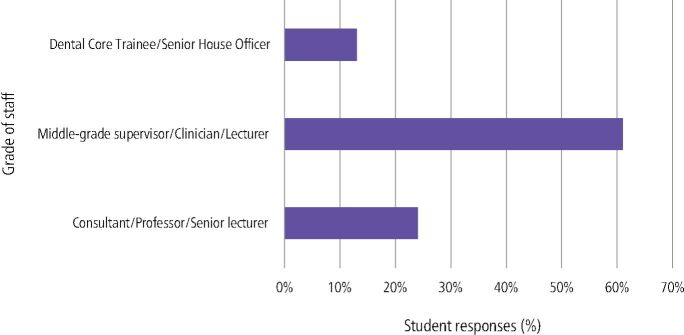
What level of staff do you think have engaged most in chairside teaching in your experience, in any clinic?

**Fig. 4:**
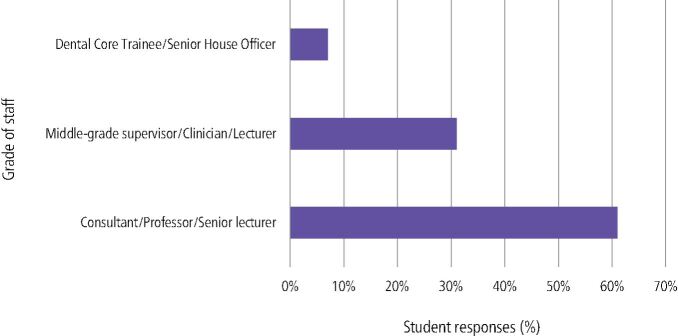
What level of staff do you think have engaged most in chairside teaching which has been related to your human disease teaching and learning?

For chairside learning in any dental topic, students reported that middle-grade supervisors engaged with them most (Fig. 3), and for chairside learning in relation to HD topics, consultant or senior lecturer staff engaged the most (Fig. 4).

### Section 3: Consolidation of learning through chairside teaching

**Fig. 5:**
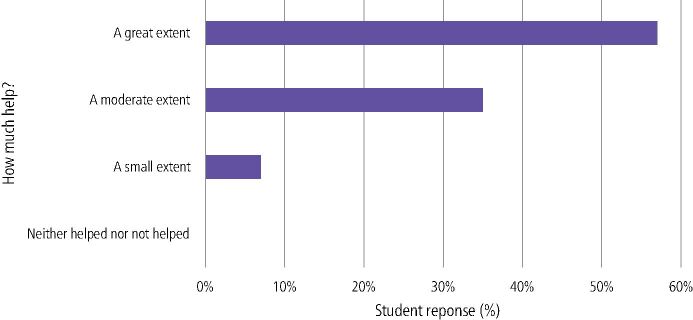
How much do you think chairside teaching helping consolidate learning in any dental topic?

In relation to any dental topic, 57% (n = 73) felt that chairside teaching had helped consolidate their learning to a great extent and 35% (n = 44) to a moderate extent. Only 7% (n = 9) felt chairside teaching had helped to a small extent (Fig. 5).

**Fig. 6:**
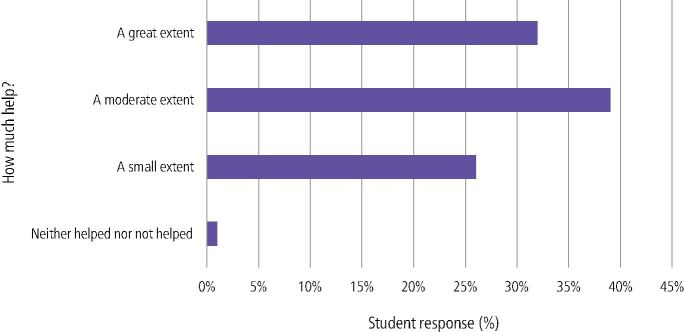
How much do you think chairside teaching helping consolidate learning in human disease topics?

In relation to HD learning, 32% (n = 41) felt that chairside teaching had helped consolidate their learning to a great extent and 39% (n = 50) to a moderate extent. Additionally, 26% (n = 33) felt chairside teaching had helped to a small extent, whilst 1% (n = 1) felt it had not helped (Fig. 6). Students gave both positive and negative free-text comments relating to this section (Table 1).

**Table 1:** Free-text comments relating to consolidation of learning through chairside teaching

Do you have any comments on this questionnaire survey or on your experiences with chairside teaching?	
Positive comments	Student year
When done well, chairside learning is excellent at putting theoretical practice into real-life perspective	5
Having teaching alongside clinical sessions cements the academic sessions we have	5
It helps hugely in applying existing knowledge to clinical situations	5
Most of my knowledge has been solidified during chairside learning opportunities	4
Chairside teaching definitely helps consolidate learning and represents a large proportion of teaching in Year 4	4
This questionnaire was very comprehensive and is a useful reflective tool. In my experience, chairside teaching is useful when it is carried out in a non-stressful environment	4
Good-quality chairside teaching would be of great help to learning in human diseases and in general	3
Chairside learning is very helpful and important	3
Chairside teaching has been useful when it has happened, but doesn't happen nearly as often as it should do	3
More chairside teaching would be nice but I understand this is not always possible as supervisors are usually very busy managing multiple students at once	3
**Negative comments**	**Student year**
Supervisors must be wary not to come across as patronising or undermining - especially in the more junior years of BDS	5
Sometimes the environment is threatening and high pressure when the supervisor demeans you and you feel the patient loses confidence in you	4
Chairside teaching is limited on DEC as the supervisor-to-student ratio is too large to have detailed discussions	3
I think we need more supervisors on DEC, particularly as I feel if they weren't spread too thin over so many students they could provide more focused chairside learning	3

### Section 4: In relation to chairside teaching, what did you think enhanced your learning?

**Fig. 7:**
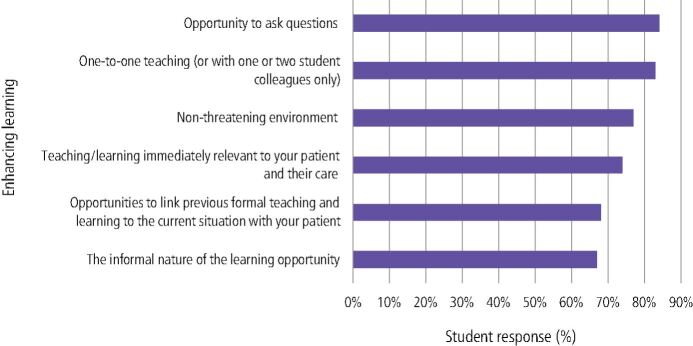
For the episodes of chairside teaching you recall in any clinic, which of the following did you think enhanced learning?

When asked to choose from a list of possible reasons that chairside teaching enhanced learning, many felt ‘the opportunity to ask questions' (84%; n = 107) and ‘one-to-one teaching' (83%; n = 106) were important. In addition, 77% (n = 98) identified ‘non-threatening environment' and ‘teaching immediately relevant to the patient and their care' (74%; n = 94) as reasons. Finally, 68% (n = 87) felt ‘opportunities to link previous formal teaching to current patient care' and the ‘informal nature of the learning opportunity' (67%; n = 85) were significant (Fig. 7).

## Discussion

Online surveys typically receive response rates of 20-47%.^11^ Our survey received an overall response rate of 62% (ranging between 58-64% for the year groups), suggesting our results are applicable to the wider student group. Reminder emails have also been shown to improve response rates.^12^

From the free-text comments, it was clear that the surveyed dental students recognised that they were engaged in chairside learning in most clinics, and that for HD topics, it was mainly in the service clinics of oral medicine, oral surgery, sedation and the dental emergency clinic. The restorative DEC clinic is where students spend most of their undergraduate clinical teaching time; therefore, it was not surprising that this was the clinic they felt they experienced most general dental chairside teaching.

The dental emergency clinic and oral surgery LA extraction clinic has a high patient turnover, with short treatment episodes, therefore providing ample opportunities for chairside teaching in both general dentistry and HD topics. The complex medical histories of patients on DEC,^8^ dental emergency and oral surgery clinics are particularly useful for learning about medicine of relevance to dentistry, and related discussions with staff are common.

In the restorative DEC clinics, the supervisors are more often experienced general dental practitioners (GDPs) who work one or two sessions a week in the dental school, or university lecturers who are former GDPs without dental specialty training. They can supervise, teach and pass on the wisdom of experience, and clearly this was recognised by the students in the form of chairside learning in relation to general dental topics.

On the dental service clinics, there will be specialists who may be consultants or senior lecturers, as well as mid-grade staff who are experienced clinicians and comprise a mix of university lecturers and NHS service delivery staff. HD learning was most recognised from consultant/professor/senior lecturer staff, who are more likely to routinely provide services within this domain, such as on oral medicine service clinics. Dental core trainees and senior house officers, who are the least experienced staff (typically between one and three years qualified), were also recognised as providing chairside teaching, and some of the free-text comments suggested that students welcomed teaching from these colleagues, who are only a few years ahead of them in their dental careers.

Sweet *et al*. (2008) reported that ‘dental tutors appeared to be enthusiastic subject specialists or practitioners who were keen to transfer their skills to the students',^13^ which mirrors the student experiences reported in this study. Similarly, Gerzina *et al*. (2005) emphasised ‘the value of providing time and resources for clinical demonstrations, faculty development in empathic skills, and the restructuring of clinical sessions to include time for discussion of clinical objectives, clinical alternatives, adequate feedback, and clinical demonstrations'.^14^

Henzi and colleagues (2005) investigated North American dental students' perspectives about their clinical education, finding that students were most appreciative of a faculty that were ‘knowledgeable and eager to help' and they noted how fortunate they were to ‘work with faculty who had a firm understanding of clinical skills and the ability to communicate these skills at the students' level of understanding'.^15^ This echoes the findings of our study where chairside teaching by enthusiastic and knowledgeable staff was greatly appreciated by the clinical dental students.

Gimson *et al*. (2019) suggested practical ways that bedside teaching should be encouraged to ensure its longevity, in a time of increasing time pressures and demands on NHS staff which often hamper such ad hoc learning opportunities.^16^ This bedside teaching in medicine is immediately analogous to chairside teaching in dental clinics, and the same levels of support and encouragement should come from dental schools to their staff as it should within medical schools.

As well as having value to those ‘being taught', bedside teaching has been shown to benefit junior doctors who provide bedside teaching,^17^ and for the patient who is the focus of the informal teaching session.^18^^,^^19^

## Conclusion

Dental students are grateful for chairside teaching/learning opportunities provided by all levels of staff in both restorative DEC and service clinics, relating to general dental topics and HD topics. Such chairside opportunities can reinforce academic learning, building upon the foundations provided in more formal teaching settings, such as timetabled lectures and tutorials. Because chairside teaching is unscheduled and relies on ad hoc opportunities for staff to engage students on clinics, it is perhaps largely unrecognised by institutions but likely deserves more appreciation and encouragement than it currently receives.

## Data Availability

Original data can be requested from the corresponding author.
